# Delaware County Medical Society

**Published:** 1856-05

**Authors:** A. E. Smith, A. Boomer

**Affiliations:** President; Secretary


*Delaware County Medical Society.*

The following named regular physicians of Delaware county, Iowa, met this day for the purpose of forming a County Medical Association, to-wit: John Acres, Albert E. Smith, Albert Boomer, Josh. F. Stout, Joshua Doran, E. C. Taylor, and James Wright.

On motion of Dr. A. E. Smith, Dr. John Acres was elected President, *pro tem.*, and Dr. A. Boomer, Secretary.

On motion of Dr. J. F. Stout, the following Constitution and By-Laws were adopted for the government of the Association:

CONSTITUTION.

ART. I. This Society shall be called the *Delaware County Medical Society.*

ART. II. All persons residing within the State of Iowa, on producing satisfactory evidence of being graduates of any regularly constituted medical college, or licentiates of any properly organized regular medical society, or passing a satisfactory examination before the Board of Censors of this Society, shall be entitled to membership on signing the Constitution and paying into the treasury one dollar.

Art. III. The object of this Society shall be, the mutual improvement of its members in the various branches of medical, surgical, and pharmaceutical knowledge.

Art. IV. The officers of this Society shall be, a President, two Vice-Presidents, Corresponding and Recording Secretaries, Treasurer, and three Censors, whose duties shall be defined in the By-Laws, and who shall be elected annually on the first Monday of June, and hold their offices until their successors are elected.

Art. V. This Society shall keep a common seal, and give to each of its members a sealed diploma of membership, signed by the President and Secretary.

Art. VI. Each member shall make an annual contribution to the fund of the Society, of not less than one dollar.

Art. VII. The Constitution may be amended at any annual meeting, by a vote of two-thirds of its members present, in favor of such amendment: *Provided*, that notice of such proposed alteration shall be given at the annual meeting preceding such alteration. The By-Laws may be amended by a majority vote at any annual meeting.

Art. VIII. The annual meeting of the Society, shall be on the first Monday of June of each year, at such place as the Society may direct; and such other meetings may be called as may be thought necessary.

Art. IX. At each annual meeting of the Society, at least two essays upon scientific subjects shall be read by such persons as may have been appointed by the President for that purpose, copies of which shall be at the disposal of the Society.

Art. X. It shall be the duty of each member to keep a record of all interesting cases that may come under his observation, and report the same at the first meeting of the Society.

Art. XI. Members may be expelled at any annual meeting, for immoral, ungentlemanly, or unprofessional conduct, provided two-thirds of the members present, vote in favor of such expulsion.

The following officers were elected for the ensuing year :—

A. E. SMITH, *President.*

ACRES & DORAN, *Vice-Presidents.*

A. BOOMER, *Secretary.*

E. C. TAYLOR, *Treasurer.*

A. E. Smith, J. Doran, and J. F. Stout, *Censors.*

*Resolved*, That the *Delhi Republican* and the *North-Western Medical and Surgical Journal* be furnished with copies of the proceedings of this meeting for publication.

A. E. SMITH, *President.*

A. BOOMER, *Secretary.*

